# On Pump Coronary Artery Bypass Graft Surgery Versus Off Pump Coronary Artery Bypass Graft Surgery: A Review

**DOI:** 10.5539/gjhs.v6n3p186

**Published:** 2014-03-24

**Authors:** Mohammad Yousuf-ul Islam, Muhammad Umer Ahmed, Muhammad Shahzeb Khan, Faizan Imran Bawany, Asadullah Khan, Mohammad Hussham Arshad

**Affiliations:** 1 Dow Medical College, Dow University of Health Sciences, Karachi, Pakistan; 2 Ziauddin Medical University, Karachi, Pakistan; 3 Cardiac Surgery Department, Civil Hospital, DUHS, Karachi, Pakistan; 4 Aga Khan University Hospital, Karachi, Pakistan

**Keywords:** coronary artery bypass graft surgery, off pump, on pump, CABG

## Abstract

There are two basic ways of performing coronary artery bypass graft surgery (CABG): on pump CABG and off pump CABG. Off pump CABG is relatively a newer procedure to on-pump CABG and does not require the use of the cardiopulmonary bypass machine. On pump CABG is the more traditional method of performing bypass surgery. However its resultant inflammatory effects cause renal dysfunction, gastrointestinal distress and cardiac abnormalities which have forced the surgeons to look for alternatives to the procedure. An extensive literature search revealed that on pump CABG causes better revascularization as compared to off pump CABG while off pump CABG has a much lower post operative morbidity and mortality especially in high risk patients. We suggest that the technique used should depend on the ease of the surgeon doing the operation as both the methods seem almost equally efficient according to the review.

## 1. Introduction

Coronary artery bypass graft surgery (CABG) has become recognized as one of the principle therapies to prolong survival and improve the quality of life of patients suffering from coronary artery disease (CAD) (Bonow & Epstein, 1985). CABG was introduced by Alexis Carrel in the early 20^th^ century when he experimented with grafting on canine models (Shumacker, 1992). His ideas were revived in the early 1960s by Konstantinov (2004), who became the first person to successfully perform the coronary artery bypass on a human being. The procedure was popularized by Favaloro, who in 1967 published the results of his successful operation on 15 patients.

Different studies have shown CABG to be the most superior method of treatment to CAD, especially in high-risk patients. In comparison to medical therapy (taking drugs like Beta blockers, nitrates etc), CABG has shown to statistically increase survival in high risk patients. Yusuf et al. (1994) reported that patients who underwent CABG had significantly lower mortality then those who opted for medical treatment after 5 years (10.2% mortality for CABG vs 15.8% mortality for medical treatment, p < 0.001), after 7 years (15.8% mortality for CABG vs 21.7% mortality for medical treatment, p < 0.001) and after 10 years (26.4% mortality for CABG vs 30.5% mortality for medical treatment, p < 0.05).

Moreover, in contrast to Percutaneoustransluminal coronary angioplasty (PTCA), CABG has shown to be a better option in the long term treatment of CAD. It was found that that patients undergoing PTCA were five times more likely to have revascularizations in 5 years succeeding treatment as opposed to patients choosing CABG (BARI, 1996). This blunted the initial cost-effectiveness of the PTCA procedure (Rihal, Raco, Gersh, & Yusuf, 2003). Serruys et al. (2009) reported that in severe cases of CAD, if patients underwent PTCA they had a significantly higher chance of encountering adverse cerebrovascular or cardiac events in the 12 months following treatment, as compared to those who underwent CABG. While Hannan et al. (2008) reported that CABG was associated with lesser deaths and myocardial infarctions as opposed to drug eluting stents over an 18 month period.

There are two basic ways of performing CABG: On pump CABG and off pump CABG. They both begin with the surgeon harvesting blood vessels from the leg, chest or the arm. The surgeon gains access to the heart using midline sternotomy. In on-pump CABG the heart is stopped with the body’s blood supply being maintained by the cardiopulmonary bypass (CPB) machine. While the heart is stopped the surgeon performs the graft procedure by sewing one end of a section of a blood vessel over a tiny opening made in the aorta and the other end over a tiny opening made in the blocked coronary vessel, distal to its blockage. With the grafting complete, the body is removed from the cardiopulmonary bypass machine and the heart is restarted (Shekar, 2006).

In off-pump CABG, the area around the blocked coronary artery is stabilized while the surgeon grafts the blood vessel on the pumping heart. Off pump CABG is relatively a newer procedure to On-pump CABG and doesn’t require the use of the cardiopulmonary bypass machine.

On pump Coronary artery bypass (ONCAB) is the more traditional method of performing bypass surgery. However its resultant inflammatory effects cause renal dysfunction, gastrointestinal distress and cardiac abnormalities which forced the surgeons to look for alternatives to the procedure (Godinho, Alves, Pereira, & Pereira, 2012).

Off pump coronary artery bypass (OFCAB) has been around since the times of Kolesov. But it gained popularity, as a variant of on-pump coronary artery bypass, due to its recently discovered effects of causing less inflammation, less morbidity and being more cost effective (Hijazi, 2010). Hence in this review we will discuss the advantages of ONCAB over OFCAB and vice versa.

## 2. Advantages of On-Pump

An extensive literature search reveals that ONCAB causes more complete revascularization as compared to OFCAB. According to Robertson and colleagues (2013), on-pump patients tend to have significantly higher frequencies of complete revascularization as compared to patients treated via Off-pump method (88.3% to 79.2%, p = 0.002). These finding are supported by Ivanov et al. (Ivanov, Borger, Tu, Rao, & David, 2008; Synnergren, Ekroth, Odén, Rexius, & Wiklund, 2008; Nakano, Okabayashi, Noma, Sato, & Sakata, 2013).

Ivanov et al. (2008) further showed that a fewer amount of distal anastomosis were formed during off-pump coronary artery bypass surgeries as compared to on-pump procedures (2.6+/- 1to 3.1 +/- 1, p < 0.001). Due to incomplete revascularization, Lattouf and colleagues (2008) have reported that patients requiring more than 3 grafts are more likely to be treated on-pump.

Incomplete revascularization has traditionally been associated with increased mortality. In 1981, Buda et al. revealed that only 69% of incompletely revascularized patients survive for the 5 year follow up as compared to 84% of completely revascularized patients. A more recent study (Jones & Weintraub, 1996) showed that incomplete revascularization correlates with decreased patient survival, in the long term, and significantly higher prevalence of recurrent angina. This body of evidence supports the argument that incomplete revascularization caused by off pump bypass surgery could lead to increased mortality in off pump bypass surgeries as compared to on-pump surgeries.

Incomplete revascularization also leads to increased repeat procedures. Hence OFCAB patients have higher rates of repeat revascularizations as compared to ONCAB patients (Ivanov et al.,2008). One study noted this difference to be quite stark when the calculated hazard ratio of OFCAB patients getting repeat procedures as compared to ONCAB came out to be 1.55 (95% CI, 1.33-1.8) (Hannan et al.,2007).

On pump bypass is preferred to off-pump bypass in emergency situations. This makes sense, as loading an already ischemic heart with additional workload by performing Off-pump surgeries seems counterproductive. Darwazah, Sham’a, Isleem, Hanbali and Jaber (2009) further showed that mean ejection fraction is significantly lower in patients going for OFCAB as opposed to those going for ONCAB (28% +/- 9% vs. 39% +/- 10%) in emergency situations. They also proved that using cardiopulmonary bypass (CPB) machines during emergency procedure lowers rates of recurrent angina, lessens symptoms of heart failure and decreases re-hospitalization frequency.

Though it was initially thought that using cardiopulmonary bypass machine increases the incidence of ischemic cerebral injury (Knipp et al.,2004), however many studies have failed to show the superiority of off-pump bypass over on-pump in avoiding cerebral injury (Puskas et al., 2003; Angelini, Taylor, Reeves, & Ascione, 2002). Indeed, Hammon and colleagues (2007) have reported that neuropsychological deficits in patients undergoing ONCAB with single cross-clamping and minimal aortic manipulation is lower than those undergoing OPCAB. They further hypothesized that mild hypothermia as obtained in on-pump surgeries could actually be beneficial for the brain.

The final advantage that on-pump procedures have over off-pump is the surgeons’ familiarity with this surgery (Légaré & Hirsch, 2006). Considering OFCAB is said to be very technically demanding and is supposed to have a prolonged learning curve, most surgeons are comfortable with using ONCAB, the time honored procedure (Lamy et al.,2012). As Brown et al. (2001) demonstrated that a surgical team’s experience and familiarity are crucial factors for a good outcome, making ONCAB slightly more superior to OFCAB in this regard.

## 3. Advantages of Off-Pump

Comparing the morbidity and mortality rate, both the off- and on-pump techniques yield very good results with very low mortality. However a much lower post operative morbidity with the off-pump operation is established. The much higher morbidity in the on-pump bypass is seemed to be much attributable to cardiopulmonary bypass process itself (Sabik et al.,2002). This is further supported by a research which shows that off-pump technique was associated with a much decreased risk-adjusted operative mortality from 2.9% with the on-pump group to 2.3% amongst the off-pump group. The adoption of an off-pump procedure also decreased the risk-adjusted complication rate from 14.15% with ONCAB to 10.62% in OFCAB (Cleveland, Shroyer, Chen, Peterson, & Grover, 2001). In short the patients who underwent OFCAB were much less likely to die and have major complications (Cleveland et al.,2001). Adding even more to this point a research highlighted that the patients who were treated off-pump had both lower complication rates (8.8% versus 14.0%) and lower mortality rates (2.7% versus 4.0%) (Plomondon et al.,2001). The aforementioned facts therefore highlight the importance of OFCAB in reducing both mortality and morbidity and lesser post operation complications.

CABG involvesa systemic inflammatory response associated with cytokine release and complement activation due to the fact that it is an invasive process. Comparing the degree of inflammatory response via the levels of pro inflammatory cytokines and the markers of inflammation in both procedures, it was revealed that the release of interleukin-8, interleukin-6, and tumor necrosis factor receptors 1 and 2 were higher in the ONCAB group than the OFCAB group (Strüber et al.,1999). Another research showed that the serum levels of tumor necrosis factor-alpha were detected in higher amounts in patients with ONCAB. Similarly, the patients in ONCAB group had more hypotension, required more inotropic drugs, had higher heart rates, higher temperature, increased postoperative bleeding, a longer orotracheal intubation time and a much more pronounced leukocytosis compared with OFCAB group (Brasil, Gomes, Salomão, & Buffolo, 1998). Therefore the much increased levels of the pro inflammatory cytokines in the ONCAB group could be the factor which causes more post op complications and mortality amongst patients.

Many studies using transcranial Doppler have shown much higher rates of cerebral vessel embolization in ONCAB patients in comparison with OFCAB patients. Majority of the studies which examined the neurocognitive function have established a little more decline amongst the ONCAB patients in comparison with OFCAB patients in the short term (<2 to 3 months) period but have failed to prove anoteworthy difference at the 1 year time period (Van Dijk etal., 2002). Another study established that ONCAB was linked to more cerebral microemboli (575 ± 278.5) as compared to OFCAB (160 ± 19.5) and lead to a significantly reduced cerebral perfusion postoperatively to the thalami, cerebellar, precunei, bilateral occipital, and left temporal lobes. However cerebral perfusion with OFCAB was found to be unchanged (Jeffrey et al., 2003). Therefore OFCAB significantly reduces the occurrence to cerebral microemboli as compared to those getting the ONCAB operation.

Comparing patients with multiple co-morbidities, 4 studies were reviewed and patients were classified as being high risk if there was the presence of multiple preoperative co morbid factors. The risk factors taken were recent myocardial infarctions, left ventricular dysfunction, left main stem disease, kidney failure, previously occurred strokes, shock, unstable angina, heart failure, chronic obstructive pulmonary disease, age greater than 70 years and urgent or emergent surgery (Bittner & Savitt, 2002; Chamberlain, Ascione, Reeves, & Angelini, 2002; Meharwal et al., 2002; Gaudino et al.,2004). A significant number of patients in the ONCAB group had 3 vessel disease, severe heart failure symptoms and unstable angina. The OFCAB patients were inclined towards having higher numbers of risk factors. However there were no significant differences to be accounted for almost all of the preoperative risk factors present. In about two researches, the number of grafts placed was found to be greater in the ONCAB group in comparison to the OFCAB group, but however the number of grafts was found to be alike in other studies. Mortality was found to be much higher in ONCAB group in one of the studies but however not much difference was found in the other 3 studies. Intensive care unit and hospital length of stay were found to be lower in the OFCAB group. In these studies, the postoperative blood loss, need for blood transfusion, arrhythmias, and ventilation time was found to be higher in the oncab group as compared to the ofcab group. According to 2 meta anlaysis the ofcab procedure significantly reduced the incidence of postopertive stroke (Afilalo, Rasti, Ohayon, Shimony, & Eisenberg, 2012; Sa et al., 2012). Similarly, peri-operative myocardial infarction was found to be lower in the OFCAB group in one study but was found not to be significantly different in the other 3 studies. However the infectious, renal, and neurological complications were found to be similar in all the studies. Therefore OFCAB can be done with a much lower mortality and morbidity in the selected groups of high-risk patients with multiple co morbid. OFCAB is a reasonable, and may even be a preferable, operative strategy for the high-risk group of patients.

Two studies were found which assessed the possibility that whether coronary revascularization surgeries in individuals with severely damaged atheromatous aorta could be performed using the off pump technique or not (Sharony et al., 2003; Sharony et al., 2004). Both the studies used the propensity matching in order to have same number of individual patients in the OFCAB and the ONCAB groups with similar preoperative characteristic and echocardiography findings of ascending aortic atheromatous disease. The incidence of stroke and mortality were found to be greater in the ONCAB groups in both the studies. The 1st study (Sharony et al.,2003) reported a stroke rate of 4.7% for the ONCAB in comparison to 2.4% for the OFCAB group and in hospital mortality rate of 11.4% and 3.8% respectively. The second study (Sharony et al.,2004) reported in hospital mortality rate of 11.4% for the traditional CABG group whilst 6.5% for the OFCAB group and a stroke rate of 5.7% and 1.6% respectively. In both the studies three years follow up was done. One study showed an increased survival in the OFCAB group whilst the other showed no significant difference. These facts therefore help reinstate that patients who had severe atherosclerotic aortic disease and underwent OFCAB show a significantly lower prevalence of complication, strokes and in hospital mortality in comparison with the patients who underwent ONCAB.

For older patients, the off pump procedure yielded better prognosis and outcome in comparison with its ONCAB counterpart. This fact is supported by 2 researches, in which one study selected patients greater than 75 years of age whilst the other study focused on patients greater than 80 years of age. However the preoperative patient characteristics were similar in both the studies. Moreover, the number of grafts placed in both the studies was found to be higher in the ONCAB group (Hoff et al., 2002; Hirose, Amano, & Takahashi, 2001). The occurrence of bleeding, transfusions, stroke, prolonged respiratory failure, and ICU and hospital length of stay were all found to be higher in the ONCAB groups in comparison to the OFCAB group. However, kidney failure, myocardial infarction, reoperation for bleeding, operative mortality and wound infections were not found to be significantly different amongst the two types of procedures. The aforementioned facts therefore help conclude that OFCAB procedure is much more beneficial when performed in elderly patients.

For patients with acute myocardial infarction a research carried in Israel highlighted the efficacy of the OFCAB procedure for such patients. The research included 225 patients who underwent CABG sometime soon after an acute myocardial infarction. All the patients who were a part of the research had similar pre operative characteristics and had the operation performed within one week after the occurrence of an acute myocardial infarction (Locker et al.,2003). The OFCAB group had more patients with one or two grafts whilst the ONCAB group had more patients with greater than three grafts. The mortality rate was significantly higher in the ONCAB group (12% compared to 3.8%), but majority of deaths in the ONCAB group were within 2 days of myocardial infarction. However the mortality rate was not significantly different in patients operated more than 2 days after myocardial infarction. The incidence of late mortality was however found to be much lower in the traditional CABG group. Therefore the use of OFCAB procedure in emergency patients being operated within the first 2 days of the onset of symptoms has found to be much beneficial in comparison to its traditional counterpart.

## 4. Conclusion

Table 1 shows a summary of the advantages of each technique. To conclude we could say that short term morbidity and mortality is less in very high-risk patients with off-pump, possibly because the procedure is shorter. It would be right to say that shorter the procedure, the better, especially for older, sicker patients. The length of the procedure is significantly shorter with off-pump than on-pump. However we suggest that the technique used should depend on the ease of the surgeon doing the operation as both the methods seem almost equally efficient according to the review otherwise. Certainly more data over large randomized trials is required before off pump superiority over on-pump can be firmly established.

**Table 1:** The main advantages of each technique

Advantages of ON Pump CABG
1) Leads to more complete revascularization
2) Leads to increased formation of distal anastomosis
3) Is a better option in emergency situations
4) Most surgeons are familiar with this form of CABG
Advantages of Off Pump CABG
1) Decreased morbidity and mortality rates
2) Associated with decreased levels of post-CABG inflammatory cytokines
3) Reduces occurrence of post-surgical cerebral microemboli
4) Preferred option in high risk patients and those with severe atherosclerotic aortic disease
5) Preferred option in older patients (i.e. > 75 years)

**Abbreviations**
CAD: coronary artery disease
CABG: Coronary artery bypass graft surgery
PTCA: Percutaneoustransluminal coronary angioplasty
ONCAB: On pump Coronary artery bypass
OFCAB: Off pump coronary artery bypass
CPB: cardiopulmonary bypass
